# Poor health status before the COVID-19 pandemic is associated with unfavourable changes in health-related lifestyle

**DOI:** 10.1177/14034948231163960

**Published:** 2023-04-12

**Authors:** Tuija Jääskeläinen, Katri Sääksjärvi, Arto Pietilä, Satu Männistö, Niina E Kaartinen, Annamari Lundqvist, Seppo Koskinen, Päivikki Koponen

**Affiliations:** Department of Public Health and Welfare, Finnish Institute for Health and Welfare, Helsinki, Finland

**Keywords:** Health-related lifestyle, COVID-19 pandemic, chronic diseases, socioeconomic status, population-based study

## Abstract

**Aims:**

The effects of COVID-19 containment measures on health-related lifestyle have been both favourable and unfavourable for health. Factors predisposing to unfavourable changes are still poorly known. In this short communication, we aimed to examine which socioeconomic and health-related factors predicted unfavourable lifestyle changes based on data from the same individuals before (2017) the pandemic and during the second wave (2020) of the pandemic in Finland.

**Methods:**

This individual-level follow-up study was based on a nationally representative, two-stage stratified cluster sample of Finnish adults from the FinHealth 2017 Study, conducted in Spring 2017, and its follow-up survey, conducted in Autumn 2020. A total of 3834 men and women aged 25–69 years at baseline had information of selected lifestyle factors (vegetable consumption, leisure-time physical activity, sleeping problems and nightmares) available at both time points. Odds ratios and 95% confidence intervals for unfavourable lifestyle changes (yes/no) according to socioeconomic and health-related factors were calculated using logistic regression models taking into account the sampling design and non-response.

**Results:**

We found that those having poor health (i.e. psychological distress, poor self-rated health or chronic diseases) or disadvantaged socioeconomic background before the pandemic were prone to unfavourable lifestyle changes during the follow-up.

**Conclusions:**

**Observed unfavourable lifestyle changes in vulnerable population groups may accelerate health inequalities. Targeted health promotion actions are needed to prevent this unfavourable development.**

## Introduction

The COVID-19 pandemic and measures aiming to control it have affected health-related lifestyle both positively and negatively. Previous findings have shown an increase in unhealthy snacking [1, 2], but the consumption of vegetables, for example, has been shown both to increase and decrease [3]. Many individuals have decreased their physical activity (PA), but also opposite changes have been reported [3, 4]. Further, the prevalence of sleep disorders has been rather high during the pandemic [5]. It is likely, however, that unfavourable lifestyle changes are not evenly distributed in the population. It is important to identify the population groups that have been particularly vulnerable to unfavourable changes.

We analysed (a) changes in the selected lifestyle factors and (b) which socioeconomic and health-related factors predicted unfavourable changes based on the individual-level follow-up data on the same individuals before (2017) the COVID-19 pandemic and during the second wave (2020) of the pandemic in Finnish adults. As predictors we focused on selected factors which may have weakened resilience during the epidemic: (a) socioeconomic factors which are known to predispose to poor health [6], and (b) indicators of poor health status at baseline. As outcomes, we focused on changes in vegetable consumption, leisure-time PA and sleeping due to relatively small changes observed in smoking and alcohol consumption during the follow-up.

## Methods

**Figure 1.**
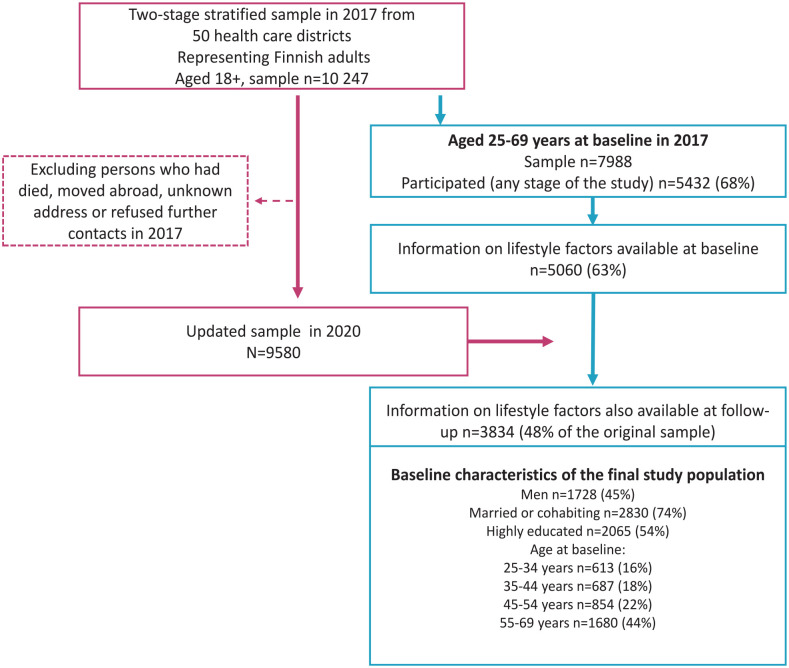
Flowchart of the study population.

The study was based on a nationally representative, two-stage stratified cluster sample of Finnish adults from the FinHealth 2017 Study, conducted in Spring 2017 [7], and its follow-up survey, conducted in Autumn 2020 [8]. The final data included a total of 3834 participants aged 25–69 years at baseline for whom information on lifestyle factors was available in both years (Figure 1). The ethics committee at the Hospital District of Helsinki and Uusimaa approved both data collections.

Lifestyle factors were measured with the same questions in both years. The consumption of vegetables was asked: ‘How often have you eaten vegetables and root vegetables (not potatoes) during the past seven days as such, grated or in fresh salads?’ with five frequency categories ranging from (1) not at all to (5) several times per day. The question concerning leisure-time PA included four categories: (1) physically inactive at leisure-time; (2) moderate everyday exercise (e.g. walking) several hours a week; (3) vigorous exercise (e.g. jogging) several hours a week; (4) competitive sports several times per week. Categories 3 and 4 were combined for the analysis. Sleeping problems and nightmares were asked about: ‘Over the past month (30 days), how often have you’: (1) had nightmares; (2) had trouble sleeping. The answer options were ‘often’, ‘sometimes’ and ‘not at all’. Unfavourable changes were defined as decreased vegetable consumption, as a change from higher intensity exercise to lower (e.g. from vigorous to moderate or from moderate to inactive) or as increased nightmares and/or sleeping problems. Favourable changes were defined in reverse.

Socioeconomic factors were asked about in the questionnaire and dichotomized: (1) living without a partner versus being married or cohabiting; (2) low education versus middle or high education; (3) unemployed, retired or other versus employed, self-employed or student; and (4) sufficiency of money to meet needs: not at all or little versus moderately, mostly or completely. Self-rated health was dichotomized into poor or rather poor or moderate versus rather good or good. Psychological distress was determined by the Mental Health Inventory (MHI-5) derived from the SF-36 scale [9]. A cut-off value of 52 or less was used to define psychological distress. The number of chronic diseases (0, 1, 2 or more) was calculated based on self-reported chronic diseases (diabetes, heart failure, coronary heart disease, myocardial infarction, stroke or cerebral haemorrhage or cerebral thrombosis, sleep apnoea, cancer, rheumatoid arthritis, other disease of the joints, back disease, depression, other psychological or mental illness, renal failure, asthma, chronic obstructive pulmonary disease) diagnosed or treated by a doctor.

The statistical analyses were carried out using Stata 16 [10] taking into account the sampling design and using inverse probability weights [11] to reduce the bias due to non-participation. Weighted, age-adjusted prevalences of changes in lifestyle were analysed by multinomial regression using predictive margins. The logistic regression model adjusted for age and the baseline level of the lifestyle factor of interest was used to estimate the weighted odds ratios and 95% confidence intervals for unfavourable lifestyle changes (yes/no) according to socioeconomic and health-related factors. In the logistic regression models, those being in the most unfavourable category of the lifestyle factor of interest at baseline were excluded (does not use vegetables at all *n*=88; physically inactive at leisure time *n*=802; often having nightmares and sleeping problems *n*=421).

## Results

**Figure 2.**
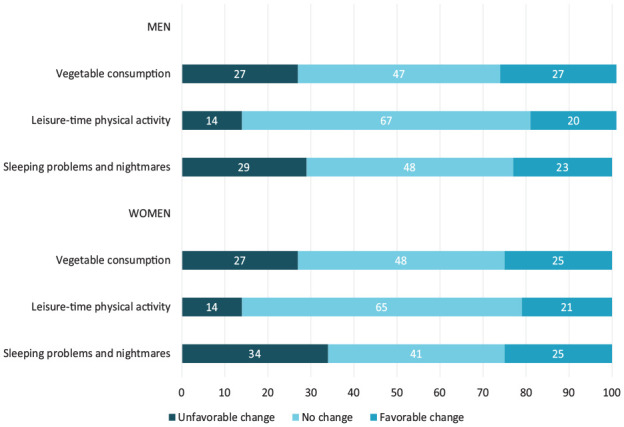
Weighted, age-adjusted prevalences (%) of changes in the selected lifestyle factors during the follow-up.

An increase in sleeping problems and nightmares was the most common unfavourable change during the follow-up in both men (29%) and women (34%), followed by a decrease in vegetable consumption (Figure 2). Further, 14% of men and women decreased their intensity of leisure-time PA. Overall, those having poor self-rated health or diagnosed chronic diseases, especially multiple chronic diseases, before the pandemic were prone to unfavourable lifestyle changes during the follow-up (Table I). Further, psychological distress before the pandemic was associated with decreased leisure-time PA in men as well as decreased vegetable consumption in women. Perceived insufficiency of money was associated with decreased leisure-time PA in both sexes as well as decreased vegetable consumption in men. Those who were not employed or studying were prone to decrease leisure-time PA. In women, living without a partner, and in men, low education, was associated with decreased consumption of vegetables.

**Table I.** Odds ratios and their 95 % confidence intervals for unfavourable lifestyle changes according to socioeconomic and health-related factors at baseline among Finnish adults aged 25–69 years at baseline.

	Living without a partner^b^	Low education^b^	Neither employed nor studying^b,c^	Perceived insufficiency of money^b^	Psychological distress^b^	Poor self-rated health^b^	Chronic diseases^b^	
							1	2+
	OR (95% CI)	OR (95% CI)	OR (95% CI)	OR (95% CI)	OR (95% CI)	OR (95% CI)	OR (95% CI)	OR (95% CI)
Men, *n* (%)^a^	385 (23)	250 (14)	332 (23)	176 (12)	98 (6)	556 (32)	484 (28)	279 (16)
Decreased consumption of vegetables^d^	1.33 (0.98, 1.81)	1.61 (1.16, 2.24)	0.93 (0.66, 1.32)	1.60 (1.07, 2.39)	1.42 (0.82, 2.47)	1.55 (1.20, 2.00)	1.04 (0.78, 1.38)	1.07 (0.75, 1.53)
Decreased intensity of leisure-time physical activity^e^	1.32 (0.88, 1.98)	1.19 (0.77, 1.85)	1.48 (1.00, 2.19)	2.15 (1.29, 3.58)	2.09 (1.13, 3.87)	1.99 (1.37, 2.89)	1.19 (0.85, 1.65)	1.53 (1.01, 2.30)
Increased sleeping problems and/or nightmares^f^	0.84 (0.61, 1.14)	0.90 (0.61, 1.33)	0.96 (0.69, 1.32)	1.25 (0.78, 1.99)	1.40 (0.69, 2.81)	1.25 (0.94, 1.68)	1.33 (1.00, 1.75)	1.67 (1.05, 2.66)
Women, *n* (%)^a^	561 (27)	223 (11)	426 (24)	226 (12)	147 (7)	572 (27)	582 (28)	344 (16)
Decreased consumption of vegetables^d^	1.41 (1.13, 1.77)	0.97 (0.67, 1.40)	0.83 (0.62, 1.10)	1.24 (0.88, 1.75)	2.02 (1.24, 3.29)	1.19 (0.91, 1.56)	1.33 (1.03, 1.72)	1.68 (1.23, 2.28)
Decreased intensity of leisure-time physical activity^e^	1.03 (0.79, 1.34)	1.34 (0.85, 2.13)	1.55 (1.11, 2.18)	1.70 (1.06, 2.73)	1.29 (0.76, 2.19)	1.80 (1.25, 2.59)	1.34 (1.00, 1.78)	1.46 (0.93, 2.29)
Increased sleeping problems and/or nightmares^f^	1.08 (0.84, 1.38)	1.05 (0.72, 1.52)	1.00 (0.76, 1.31)	1.06 (0.71, 1.59)	1.62 (0.99, 2.65)	1.42 (1.09, 1.84)	1.29 (1.02, 1.62)	1.39 (0.99, 1.95)

Logistic regression analysis adjusted for age (continuous) and baseline level of lifestyle factor of interest.

OR: odds ratio; CI: confidence interval.

a Crude values at baseline: the number of missing values ranged from 1 to 84, perceived sufficiency of money, the number of missing values was 296 in men and 249 in women.

b Reference categories: being married or cohabiting; middle or high education; employed, self-employed or student; moderately, mostly or completely enough money to meet the needs; no psychological distress, Mental Health Inventory (MHI-5) >52; rather good or good self-rated health; none of the enquired chronic diseases.

c Aged 25–64 years at baseline.

d Those who were not consuming vegetables at all at baseline (total *n*=88; men *n*=62; women *n*=26) were excluded in the analysis.

e Physically inactive at leisure-time at baseline (total *n*=802; men *n*=361; women *n*=441) were excluded in the analysis.

f Those often having nightmares and sleeping troubles at baseline (total *n*=421; men *n*=140; women *n*=281) were excluded in the analysis.

## Discussion

This population-based, individual-level follow-up study showed that lifestyle changes have been complex, being both favourable and unfavourable for health. Those having poor health status before the pandemic were prone to unfavourable lifestyle changes during the follow-up. Furthermore, a disadvantaged socioeconomic background was associated with unfavourable changes.

Our results showing a quite high proportion of adults with unchanged habits as well as changes occurring in both directions are in line with previous studies conducted in Europe [2–4]. Restrictions, recommending remote work, social isolation, and stress [12] during the epidemic may have contributed to lifestyle changes. In previous research, especially mental health problems, but also poor self-rated health, for example, have been associated with unfavourable changes [4, 13]. The present study confirmed the urgent need to target health promotion actions to vulnerable population groups with poor health status to prevent the acceleration of health inequalities. If these unfavourable changes remain permanent, and especially if they accumulate in the same individuals, they may have long-term effects on public health and increase the burden on healthcare systems.

The major strength was that this study was based on representative, follow-up data. Further, identical questions were used at both time points in the analysis of lifestyle changes. As to limitations, non-response may weaken the representativeness of the data, but we used inverse probability weights [11] to minimize this bias. Finally, our baseline data were collected in 2017, which limits the possibilities to distinguish between the impact of the pandemic versus other factors on lifestyles.

In conclusion, our results suggest that those who were already disadvantaged before the pandemic were prone to unfavourable lifestyle changes during the follow-up ending at the time of the second wave of the pandemic in Finland. Targeted health promotion actions are needed to prevent this unfavourable development. Future research is needed to examine the long-term effects of the pandemic on public health.
